# Phagocytosis and intracellular killing of *Pasteurella multocida* B:2 by macrophages: A comparative study between buffalo and cattle

**DOI:** 10.14202/vetworld.2022.275-280

**Published:** 2022-02-10

**Authors:** Qistina Hasnan, Yulianna Puspitasari, Sarah Othman, Mohd Zamri-Saad, Annas Salleh

**Affiliations:** 1 Department of Veterinary Laboratory Diagnosis, Faculty of Veterinary Medicine, Universiti Putra Malaysia, 43400, Serdang, Selangor, Malaysia; 2 Department of Veterinary Microbiology, Faculty of Veterinary Medicine, Universitas Airlangga, East Java, 60115, Indonesia; 3 Department of Cell and Molecular Biology, Faculty of Biotechnology and Biomolecular Sciences, Universiti Putra Malaysia, 43400, Serdang, Selangor, Malaysia; 4 Laboratory of Sustainable Animal Production and Biodiversity, Institute of Tropical Agriculture and Food Security, Universiti Putra Malaysia, Serdang 43400, Selangor, Malaysia

**Keywords:** buffalo, cattle, *in vitro* efficiency, macrophages, *Pasteurella multocida* B:2

## Abstract

**Background and Aim:**

*Pasteurella multocida* B:2 is the causative agent of hemorrhagic septicemia (HS) in buffalo and cattle. Buffaloes are known to be more susceptible to HS than cattle, but the reason for this remains unknown. This study aimed to compare the *in vitro* efficiency with which buffalo and cattle macrophages can kill *P. multocida* B:2.

**Materials and Methods:**

Monocyte-derived macrophages of buffalo and cattle were used in this study. They were exposed to 1×10^6^ colony-forming unit/mL of live *P. multocida* B:2 before the cells were harvested at 0, 30, 60, and 120 min post-exposure and viewed under a fluorescence microscope to count viable and non-viable macrophages and the macrophages with phagocytosing *P. multocida* B:2 cells. The phagocytosis, intracellular bacterial killing, and macrophage death rates were calculated and compared between the two species and sampling points.

**Results:**

In general, the rates of phagocytosis, intracellular killing, and macrophage death increased with time of exposure for both animal species. No significant (p>0.05) differences were noted between the phagocytosis rates by the macrophages of buffalo and cattle throughout the experiment. However, the rates of intracellular killing were significantly (p<0.05) higher in cattle macrophages at 30 min and 120 min post-exposure than those of buffalo. The death rates of buffalo macrophages were significantly (p<0.05) higher than those of cattle at 60 min and 120 min post-exposure.

**Conclusion:**

With higher bacteria killing ability and lower macrophage death, cattle appeared to be more efficient at handling *P. multocida* B:2 infection than buffalo.

## Introduction

*Pasteurella multocida* B:2 is a Gram-negative bacterium that causes hemorrhagic septicemia (HS) in buffalo and cattle. It is often associated with disease outbreaks with high lethality, leading to severe economic losses [1,2]. Although it is a septicemic disease, the respiratory tract was confirmed as the most important route of infection [3]. The pathogen enters the upper respiratory tract and translocates into the lower respiratory tract through the respiratory airway. In the lungs, it multiplies rapidly and leads to severe pneumonia and endotoxemia. Pneumonia is characterized by severe pulmonary hemorrhages, congestion, and infiltration of macrophages and neutrophils.

Although both buffalo and cattle are susceptible to infection by *P. multocida* B:2, it was epidemiologically and experimentally proven that buffaloes are significantly more susceptible to HS than cattle [4,5]. Although the reasons for this are currently unknown, the previousstudy suggested that immune-physiological differences in the respiratory tract between these two species could play a significant role [5]. In the lungs, cells that contribute to the pulmonary immune system include the aggregated lymphocytes known as bronchus-associated lymphoid tissue [6,7], and macrophages consisting of alveolar macrophages and monocyte-derived macrophages [8]. The lymphocytes contribute to respiratory mucosal immunity through the production of immunoglobulins [9], while the macrophages function in phagocytosis, antigen presentation, production of cytokines, and progression of inflammation [10,11]. The pathogenesis of HS involves the death of many macrophages, mainly due to the effect of endotoxin produced by *P. multocida* B:2 [12-14].

The infiltration of macrophages into the lungs of buffalo and cattle with HS has previously been described. The observation of *P. multocida* B:2 antigen in the cytoplasm of macrophages of carrier animals suggests that these immune cells play a key role in HS [15], perhaps in determining the outcome of the host-pathogen interactions. The results from this comparative study could provide a significant explanation of the difference in susceptibility between these two species and information that could be used to design a preventive protocol against this disease in the future. This could include the manipulation of different cytokines to increase the efficiency with which macrophages of both species can eliminate *P. multocida* B:2 and also the prevention of multiorgan failure due to septicemia.

This study aimed at evaluating and comparing the *in vitro* efficiency with which buffalo and cattle macrophages can act against *P. multocida* B:2 to explain the difference in susceptibility of these two species to HS.

## Materials and Methods

### Ethical approval

All procedures used in this study were approved by the Institutional Animal Care and Use Committee of Universiti Putra Malaysia (UPM/IACUC/AUP-R087/2019).

### Study period and location

This study was conducted from June 2020 to June 2021. Experimental animals were housed at Ruminant Research Unit of the Faculty of Veterinary Medicine, Universiti Putra Malaysia (UPM) located at latitude 3.006582, and longitude 101.705998. All *in-vitro* study was conducted at Histopathology Laboratory, Faculty of Veterinary Medicine, UPM.

### Animals

Three healthy buffaloes and three healthy cattle calves of 9 months of age with no history of vaccination against HS were selected for this study. The animals were kept in individual pens and fed with cut Napier grass at the rate of 4 kg/animal/day and supplemented with commercial feed at 500 g/animal/day. Drinking water was provided *ad libitum* to all animals.

### Preparation of inoculum

Wild-type *P. multocida* B:2 used in this study had previously been isolated from an outbreak of HS in Malaysia. The bacteria were kept at −80°C, thawed, and inoculated intraperitoneally into a mouse to attain full virulence. The bacteria were then confirmed as *P. multocida* B:2 using polymerase chain reaction, in accordance with an established method [16].

Next, the bacteria were cultured on blood agar at 37°C for 24 h before four uniformly sized colonies were seeded into Brain-Heart Infusion broth. This broth was incubated at 37°C and shaken at 150 rpm for 18 h. The bacterial concentration was routinely estimated using a serial dilution method, and the bacterial concentration was adjusted to an infective dose of 1×10^6^ colony-forming unit (CFU)/mL of live *P. multocida* B:2. The solution was then resuspended in Roswell Park Memorial Institute medium (RPMI) 1640 (Gibco, USA) and used as an inoculum. The inoculum was freshly prepared before infection.

### Peripheral blood monocyte isolation and macrophage culture

Monocytes were harvested in accordance with previously reported protocols [12,17]. Briefly, 30 mL of blood was collected into ethylenediaminetetraacetic acid (EDTA) tube, diluted with Hank’s Balanced Salt Solution at a ratio of 1:1, layered onto 3 mL of Ficoll-Paque Polymorphprep™ Plus (Cytiva, Uppsala, Sweden) and centrifuged at 600× *g* for 35 min at 4°C. The peripheral blood mononuclear cells (PBMC) were directly collected from the interface between Ficoll and the plasma-medium layer. The PBMC were then layered onto 3 mL of cold hyperosmotic Percoll™ (Cytiva) and centrifuged at 580× *g* for 15 min at 4°C. A monocyte layer that appeared between Percoll and plasma-medium layers was collected, mixed with lysis buffer, and kept at 4°C for 10 min. The suspension was then resuspended gently before centrifugation at 400× *g* and 4°C for 15 min. Following centrifugation, the supernatant was discarded and the pellet was resuspended in 1 mL of RPMI 1640 medium (Gibco, USA). A drop of the cell suspension was used to prepare a smear on a glass slide and was subjected to Wright’s staining to confirm the morphology and perform a cell purity test [18]. Trypan blue exclusion test [19,20] was applied to determine the viability of the monocytes using a standard hemocytometer (Hausser Scientific, USA).

The harvested monocytes were incubated with a maintenance medium that was prepared as follows: 5 mL of RPMI 1640 mixed with 20% fetal bovine serum and 1% antibiotic–antimycotic. The harvested monocytes were seeded in a T25 culture flask (SPL Life Sciences Co., Ltd, Gyeonggi-do, Korea) at 1×10^6^ cells/mL and incubated at 37°C in a CO_2_ incubator (N-Biotek, Gyeonggi-do, South Korea). After 24 h, non-adherent cells were discarded and 5 mL of maintenance medium was added to each flask. The maintenance medium was changed every 3 days. The adherent cells were maintained in the CO_2_ incubator for 21 days to allow them to mature into macrophages. For harvesting, the cells were incubated with trypsin-EDTA in the CO_2_ incubator for 15 min and a cell scraper was used to detach the cells. Harvested macrophages were resuspended in RPMI 1640 for further use.

### Evaluation of phagocytosis, intracellular killing, and death rates

The efficiency of macrophages was evaluated based on the phagocytosis rates, intracellular killing of bacteria, and death of macrophages. These variables were calculated through previouslydescribed formulas [12,17]. Briefly, the macrophage cultures from each species were further divided into two major groups. One group was exposed to live *P. multocida* B:2, while the other was not exposed but was kept only in the maintenance medium as a negative control.

Approximately 200 μL of the macrophage solution (concentration of 1×10^5^ macrophages/mL) was pipetted into chamber slides and incubated for 45 min to allow attachment. The cultures were then inoculated with 200 μL of live *P. multocida* B:2 inoculum containing 1×10^6^ CFU/mL, resulting in a multiplicity of infection of 10. The cells were sampled at 0, 30, 60, and 120 min post-exposure. The negative control groups were inoculated with 200 μL of sterile maintenance medium and sampled at the same intervals.

Following samplings, the macrophages were stained with acridine orange for 45 s and counter-stained with crystal violet for 1 min. The cells were then examined under a fluorescent microscope (Nikon Eclipse Ti, Japan), with a total of 100 macrophages being examined to determine the presence of viable or non-viable macrophages and *P. multocida* B:2 based on the change of color. Viable cells appeared green while dead ones appeared orange. The phagocytosis, intracellular killing, and death rates were calculated as follows:


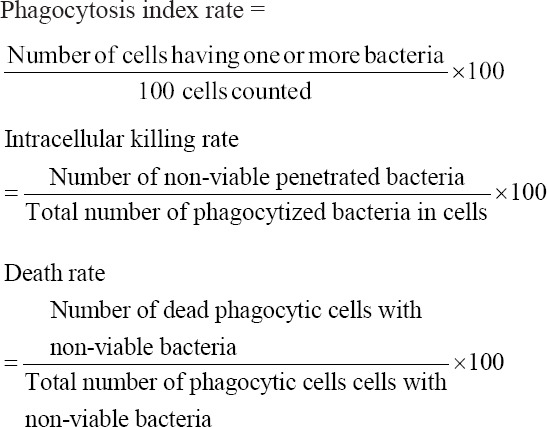


### Statistical analysis

Data on phagocytosis, intracellular killing, and death rates of macrophages were analyzed by one-way analysis of variance with *post hoc* Tukey honestly significant difference for between-group and within-group comparisons. Statistical significance was set at p<0.05. All data are presented as mean±standard deviation. The phagocytosis, intracellular killing, and death rates of macrophages were compared between buffalo and cattle at different time points. All data were analyzed using Statistical Package for the Social Sciences Statistics Version 22 software (IBM Corp., NY, USA).

## Results

**Figure-1:**
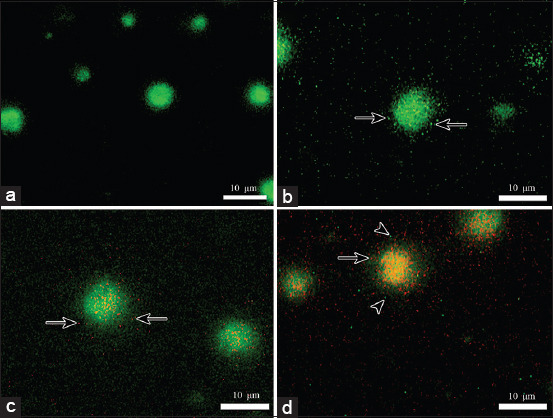
Macrophages of buffalo or cattle following exposure to *Pasteurella multocida* B:2 (acridine orange, bars = 10 μm). (a) Viable macrophage at 0 min appears green with the absence of bacterial cell. (b) Viable macrophage with phagocytosis but the bacterial cells are viable (arrows). (c) Viable macrophage with phagocytosis but with orange non-viable bacteria (arrows). (d) Orange non-viable macrophage with phagocytosed non-viable bacteria (arrow) and few green, viable bacteria (arrowheads).

In general, the phagocytosis, intracellular killing, and death rates in groups exposed to *P. multocida* B:2 showed increasing trends with increasing exposure time. At 0 min, the macrophages of buffalo and cattle did not show any phagocytic activity (Figure-1a). However, phagocytosis, intracellular killing of *P. multocida* B:2, and death of macrophages were observed starting from 30 min, which lasted until the end of the experiment (Figure-1b-d). No buffalo or cattle macrophages from the negative control group showed phagocytosis, bacterial killing, or death.

### Phagocytosis

No significant difference (p>0.05) in the phagocytosis rates between the macrophages of buffalo and cattle was observed throughout the study period. As expected, there was no phagocytosis by the macrophages of both species at 0 min. However, there were significant (p<0.05) increases in the rates of phagocytosis at 30 min post-exposure in both species, with a slightly lower (p>0.05) rate by buffalo macrophages (98.37±1.50%) than that by cattle macrophages (98.70±2.2%). At 60 and 120 min, both exposed species showed a rate of phagocytosis of 100%, which was higher, albeit not significantly (p>0.05), than the rates at 30 min post-exposure (Table-1).

**Table 1:** *In vitro* rates of phagocytosis of *P. multocida* B:2 (mean±SD) by the macrophages of buffalo and cattle.

Animal species	Treatment	Phagocytosis rate of macrophages (%) (mean±SD)			

		0 min	30 min	60 min	120 min
Cattle	*P. multocida* B:2	0.0±0.0^a^	98.7±2.2^a^	100±0^a^	100±0.0^a^
	Control	0.0±0.0^a^	0.0±0.0^b^	0.0±0.0^b^	0.0±0.0^b^
Buffalo	*P. multocida* B:2	0.0±0.0^a^	98.37±1.5^a^	100±0^a^	100±0.0^a^
	Control	0.0±0.0^a^	0.0±0.0^b^	0.0±0.0^b^	0.0±0.0^b^

^a,b^ Different superscripts indicate significant difference (p<0.05) between groups at different time points. Data represent the means (±SD) of three independent assays with triplicate samples. *P. multocida=Pasteurella multocida*, SD=Standard deviation

### Intracellular killing

There was no observable intracellular killing by the macrophages of both species at 0 min as no phagocytosis was observed at this time point. Between 30 and 120 min, the rates of intracellular killing by cattle macrophages remained significantly (p<0.05) higher than those of buffaloes. The highest rates of intracellular killing were observed at 120 min, with 58.3% for cattle macrophages and 52.39% for buffalo macrophages. Throughout the period between 30 and 120 min, the intracellular killing rates were consistently higher than the rates of control groups. Table-2 summarizes the rates of intracellular killing of *P. multocida* B:2 by the macrophages of buffalo and cattle.

**Table 2:** *In vitro* intracellular killing rates of *P. multocida* B:2 (mean±SD) by the macrophages of buffalo and cattle.

Animal species	Treatment	Intracellular killing rate of macrophages (%) (mean±SD)			

		0 min	30 min	60 min	120 min
Cattle	*P. multocida* B:2	0.0±0.0^a^	40.73±2.4^a^	51.60±6.3^a^	58.30±1.1^a^
	Control	0.0±0.0^a^	0.0±0.0^c^	0.0±0.0^b^	0.0±0.0^c^
Buffalo	*P. multocida* B:2	0.0±0.0^a^	30.92±1.7^b^	46.45±6.1^a^	52.39 ± 0.94^b^
	Control	0.0±0.0^a^	0.0±0.0^c^	0.0±0.0^b^	0.0±0.0^c^

^a,b,c^ Different superscripts indicate significant difference (*p*<0.05) at different time points. Data represent the means (±SD) of three independent assays with triplicate samples. *P. multocida=Pasteurella multocida*, SD=Standard deviation

### Death rate of macrophages

Dead macrophages were observed starting from 30 min post-exposure in both buffalo and cattle, but the differences compared with the rates at 0 min were not significant (p>0.05). Thereafter, the macrophage death rates showed a pattern of increase with increasing time of exposure.

At 30 min, the death rate was higher, albeit not significantly (p>0.05), in buffalo (3.70%) than in cattle (0.60%). On the other hand, significantly increased (p<0.05) death rates were observed at 60 and 120 min when the death rates of buffalo macrophages were 2.1-3.7 times higher than those of cattle (Table-3).

**Table 3:** Death rates (mean±SD) of buffalo and cattle macrophages following *in vitro* exposure to *P. multocida* B:2.

Animal species	Treatment	Death rate of macrophages (%) (mean±SD)			

		0 min	30 min	60 min	120 min
Cattle	*P. multocida* B:2	0.0±0.0^a^	0.6±0.5^a^	2.12±0.6^b^	4.50±0.6^b^
	Control	0.0±0.0^a^	0.0±0.0^a^	0.0±0.0^b^	0.0±0.0^b^
Buffalo	*P. multocida* B:2	0.0±0.0^a^	3.7±6.4^a^	4.49±1.7^a^	16.72±5.74^a^
	Control	0.0±0.0^a^	0.0±0.0^a^	0.0±0.0^b^	0.0±0.0^b^

^a,b^ Different superscripts indicate significant difference (p<0.05) at different time points. Data represent the means

(± SD) of three independent assays with triplicate samples. *P. multocida=Pasteurella multocida,* SD=Standard deviation

## Discussion

The results of this study revealed that macrophages of cattle more efficiently dealt with *P. multocida* B:2 infection than those of buffaloes. Although the phagocytosis rates were similar, the cattle macrophages were more efficient at performing intracellular killing. Furthermore, the rates of macrophage death were consistently lower in cattle than those in buffaloes, affecting the susceptibility of these animal species to infection by *P. multocida* B:2. These findings are interesting because it is expected that a high rate of phagocytosis and killing of *P. multocida* would lead to the release of endotoxin, subsequently leading to the death of more macrophages. This observation could be explained by the differences in the macrophage phenotypes after infection between buffalo and cattle. However, further study is needed to confirm this.

Interactions between macrophages and pathogens and the influence of the microenvironment determine macrophage phenotypes [11]. One way of determining the phenotype of a macrophage is by determining its polarization. Macrophage polarization is a process by which a macrophage adopts different functions. Non-activated M0 macrophages can be classically activated into M1 macrophages, which are further subdivided into M1a and M1b macrophages [21,22]. These M1 macrophages have a great ability to kill bacterial pathogens and secrete high levels of pro-inflammatory cytokines. Alternatively, non-activated M0 macrophages can be activated into M2 macrophages, which mainly function in tissue healing and the secretion of anti-inflammatory cytokines [23,24].

Interactions between macrophages and Gram-negative bacteria and their endotoxin typically lead to M1 polarization. It has been reported that exposure to *P. multocida* B:2 leads bovine macrophages to secrete TNF-α, which constitutes evidence of M1 polarization [25]. The consistent observations of efficient killing and lower death rates of cattle macrophages than those of buffalo macrophages as observed in this study were possibly due to faster and higher rates of M1 polarization by cattle than buffalo macrophages. Alternatively, there may be differences in the basal macrophage phenotype between buffalo and cattle, given that this phenotype is known to contribute to different outcomes after bacterial infection [26].

Apart from polarization, the different death rates of buffalo and cattle macrophages could be explained by their undergoing different types of cell death. However, the staining and parameters measured in this study could not be used to determine the mechanism of macrophage death. Nevertheless, cell death can be divided into two types: Unprogrammed (necrosis) and programmed cell death. There are many different types of programmed cell death, such as apoptosis, pyroptosis, and necroptosis, which are commonly observed in septic diseases [27]. In our previous study involving the exposure of buffalo endothelial cells to *P. multocida* B:2, it was observed that many endothelial cells showed ultrastructural changes involving membrane blebbing [13], suggestive of programmed cell death by pyroptosis. Pyroptosis tends to occur rapidly [27] and incites severe inflammatory reactions. This fits well with the fact that buffaloes are more susceptible to infection by *P. multocida* B:2, show more severe lesions, and tend to succumb faster to this infection than cattle [5]. It is thus likely that the death of macrophages in this study involved pyroptosis. A study comparing the outcome of infection by *Staphylococcus aureus* in susceptible and resistant mice concluded that the levels of pyroptosis and the pro-inflammatory cytokine IL-1B were higher in susceptible mice. However, the production of other pro-inflammatory cytokines such as TNF-α IL-6, and CXCL was suppressed [25].

### Limitations of the study

The immune response toward *P. multocida* B:2 can differ among different animals despite them being from the same species. The underlying conditions or diseases of the animals before the start of this study may also have affected the results. This was an *in vitro* study on macrophages, other immune cells were removed and only macrophages were present to eliminate the pathogens. However, given that all components of the immune system play important roles in fighting off pathogens, the presence or absence of other immune cells might lead to different results regarding not only the elimination of *P. multocida* B:2, but also the eventual fate of the animals.

## Conclusion

This study reveals interesting findings that explain the difference in susceptibility of buffalo and cattle to HS, while also raising many questions, particularly about macrophage phenotypes and the mechanisms of macrophage death. For septicemia to develop, *P. multocida* B:2 needs to translocate from the lungs into the blood vessels. In this process, interactions with different cells such as pneumocytes and endothelial cells would occur [28]. Besides macrophages, these two cell types might also play roles in the different levels of susceptibility to HS between these two ruminants. Future studies should also focus on performing macrophage phenotyping in HS and the modulation of macrophages. Information on this might be useful for the future prevention of respiratory and septicemic diseases.

This *in vitro* study reveals that macrophages of buffaloes and cattle have different levels of efficiency in terms of their responses to infection by *P. multocida* B:2. Although the rates of phagocytosis by macrophages of buffalo and cattle are similar, the macrophages of cattle show significantly more efficient intracellular killing and resistance to death than those of buffaloes. Further investigation is needed to obtain a deeper understanding of macrophage phenotypes and mechanisms of macrophage death, which could further explain the differences between the susceptibility of buffaloes and cattle to HS.

## Authors’ Contributions

AS, MZ, and SO: Conceptualization, Writing - review and editing. QH, YP, and AS: Investigation. QH and YP: Writing - initial draft preparation. All authors have read and agreed to the published version of the manuscript.
